# Variability of the Indonesian Throughflow in the Makassar Strait over the Last 30 ka

**DOI:** 10.1038/s41598-018-24055-1

**Published:** 2018-04-09

**Authors:** Weijia Fan, Zhimin Jian, Zhihui Chu, Haowen Dang, Yue Wang, Franck Bassinot, Xiqiu Han, Yeping Bian

**Affiliations:** 1 Key Laboratory of Submarine Geosciences, Second Institute of Oceanography, State Oceanic Administration, Hangzhou, 310012 China; 2 State Key Laboratory of Marine Geology, Tongji University, Shanghai, 200092 China; 3 Laboratoire des Sciences du Climat et de l’Environnement, UMR 8212 CEA-CNRS-UVSQ (LSCE), Gif-sur-Yvette, 91198 France; 4 Shanghai Tech University, Shanghai, 201210 China

## Abstract

The hydrological characteristics, including temperatures and salinities, of the upper water over the last 30 ka from two sites connected by the Indonesian Throughflow (ITF) across the Makassar Strait are reconstructed and compared. The thermocline hydrological gradient in the strait was larger during 13.4~19 ka BP and 24.2~27 ka BP than that in the Holocene. The weakened ITF during those periods in the last glacial period, corresponding to the decreased trade wind stress under an El Niño-like climate mean state, likely accounts for the increased thermocline gradient. The thermocline water temperature variabilities of the two sites, in particular the highest peaks at ~7 ka BP, are different from the records of the open western Pacific. Reoccurrence of the South China Sea Throughflow and thus a decreased surface throughflow along the Makassar Strait perhaps led to a warmer peak of thermocline temperature at ~7 ka BP than at ~11 ka BP.

## Introduction

As one of the key parts of the global thermohaline circulation (THC), the Indonesian Throughflow (ITF) originates from the warmest region of the western-Pacific Warm Pool (WPWP) and transports considerable heat to the Indian Ocean^1–3^ (Supplementary Fig. S1). Variability of the ITF brings about significant air-sea dynamic responses and is considered to be one of the most important factors of global climate change, e.g., the slowdown in surface temperature warming since 1998^4–8^. Understanding the past changes of the ITF is crucial to reveal the underlying mechanisms controlling the glacial-interglacial climatic transition in the Indo-Pacific region.

**Figure 1:**
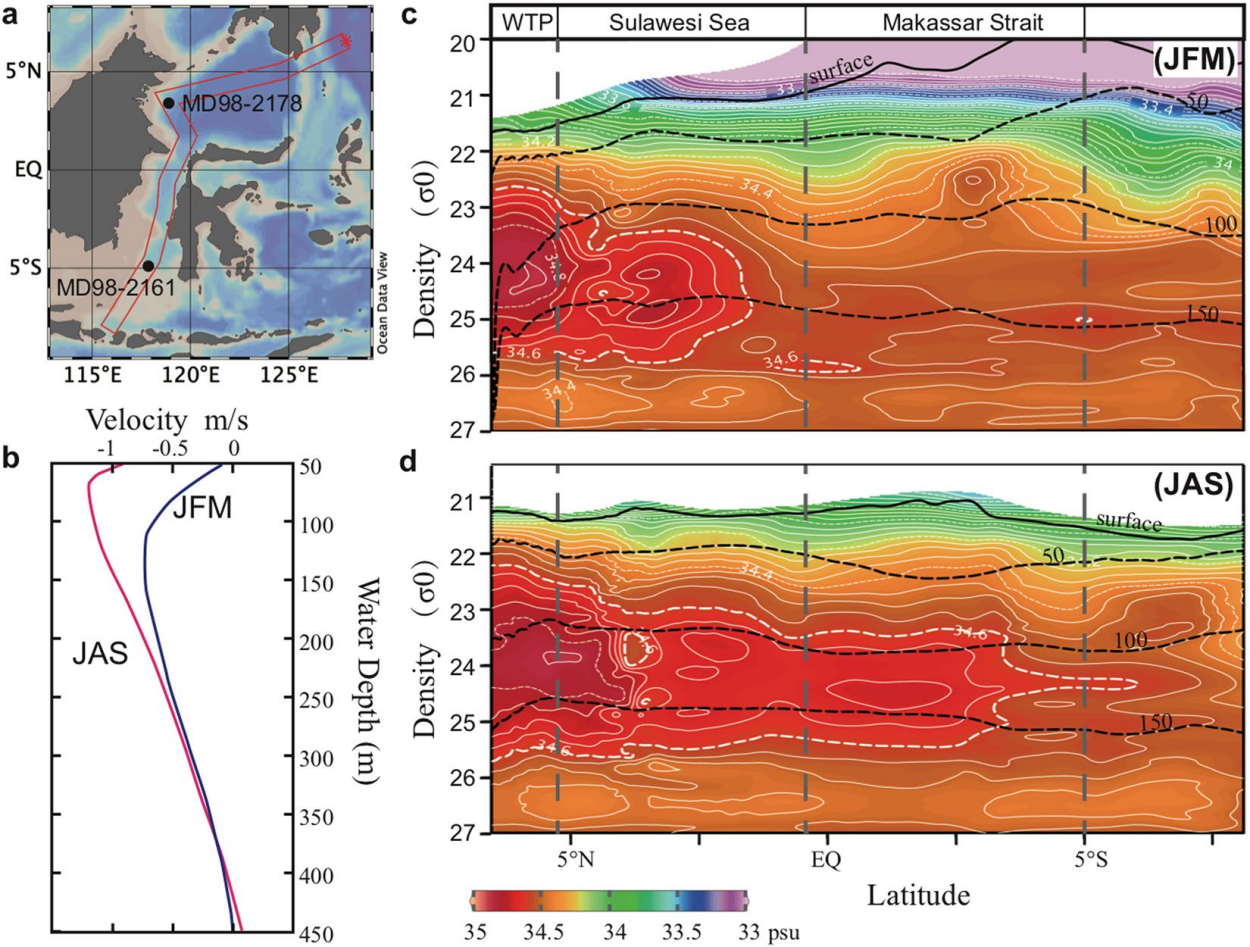
(**a**) Site locations (black dot) and the ITF transect (red rectangular); (**b**) Vertical distribution of modern ITF velocity during boreal winter (JFM, blue line) and summer (JAS, red line) with negative values standing for stronger and shallower transport during JAS than JFM; (**c**) and (**d**) are density-latitude profiles of salinity (colour shaded) along the ITF transect during JFM and JAS, respectively. Black contours in (**c**,**d**) denote water depth. White contours of the 34.6 psu salinity in (**c**,**d**) clearly show the intrusion of high salinity subsurface water from the western Pacific between 100 m and 150 m water depth, indicating a stronger ITF inflow during summer (JAS) than winter (JFM). The salinity data used are based on the World Ocean Atlas 2013 dataset^51^ and are displayed using Ocean Data View version 4.7.3^52^.

Today, the ITF inflows into the Indonesian Seas through two paths and the most important of which is the western path (Supplementary Fig. S1), in which the north Pacific water is drained into the Indonesian seas through the south cape of Mindanao Island and Sulawesi Sea, and penetrates the Makassar Strait in the form of Makassar Strait throughflow (MSTF)^9^. The MSTF is a jet-like current with a subsurface concentrated mass transport at the depth of 70~240 m and a maximum rate larger than 1 m s^−1^ (Fig. 1)^10^, and waters below 680 m cannot be transported to the Banda Sea due to topographic sills^11^. According to mooring observations, mass transport increases with heat transport across the Makassar Strait during the southeast monsoon seasons (boreal summer, represented by July, August and September which is abbreviated as JAS), because the depth of velocity maximum usually shoals and surface warm water can be more effectively transported^9,12^ (Fig. 1). When the northwest monsoon prevails during boreal winter (represented by January, February and March which is abbreviated as JFM), surface transport of the MSTF decreased due to the restriction of the northward buoyancy gradient built by the low salinity cap occupying the southern Makassar Strait^13^, while warmer and less salty surface water from the South China Sea is injected into the southern Makassar Strait through the Karimata Strait (called the South China Sea Throughflow, SCSTF, see Supplementary Fig. S1)^4,11,14,15^. From inter-annual to multi-decadal time scales, variability of the MSTF mass transport is essentially controlled by the zonal wind stress above the tropical Pacific and thus the pressure gradient between the western Pacific and eastern Indian Ocean^3^. That’s why the modern ITF is dominated by the ENSO variability and the climate mean state over the tropical Pacific, according to modern observations and ITF-related modelling studies^16–20^.

The heat transport ability of the MSTF is not only associated with its discharge flux but also with the thermal structure of those advected water masses^21^. Today, the Indonesian sea is the only region in the world where strong internal tides remain trapped in the semi-enclosed seas and thus the trapped tidal energy is available for vertical mixing^22,23^. The mixing plays an important role in re-shaping the vertical hydrological structure of the in-flowed Pacific Water^24^. Along the ITF main route, the mixing of surface and intermediate fresher waters with saltier Pacific tropical water makes the salinity maximum attenuated and finally the out-flowed ITF water is characterized by a fresher and cooler thermocline and an isohaline layer^22^. The Sulawesi Sea water can keep high salinity characteristic, but its subsurface salinity maximum is gradually attenuated along the Makassar Strait and disappears before it gets into the Banda Sea (Fig. 1). The subsurface temperature is cooler and the depth of the thermocline is much shallower in the southern Makassar Strait than in the north (Supplementary Fig. S2).

Due to the importance of the ITF on global heat redistribution, many studies were devoted to clarify how it evolved in the past. During the last glacial maximum (LGM), owing to drastic sea level drop by ~120 m^25^, the ITF should have experienced important changes in its routes, strength, thermal structure and associated heat transport ability^26^. Previous studies proposed that the ITF might have been weaker during the LGM^27–29^. Paleo-proxies of upper ocean thermal structure over the Timor Sea indicated an intensification of cooler thermocline outflow of the ITF since the early Holocene due to the reoccurrence of the SCSTF and 5 intervals with weaker thermocline outflow in the last two glacial cycles^30–32^. But these works mainly focus on the ITF outflow regions (i.e. Timor Passage), and little attention is paid to the inflow of the ITF, especially to the MSTF that takes 70~80% of the total mass transport of the ITF inflow. And notably, little is known about the variability of thermal structure and velocity profile of the MSTF that may directly determine the heat redistribution via the ITF^21,24^.

In this study, two Calypso cores MD98-2161 (MD2161, 5.21°S, 117.48°E, water depth 1185 m) and MD98-2178 (MD2178, 3.62°N, 118.70°E, water depth 1984 m) were retrieved from the south and north ends of the Makassar Strait during the IMAGES IV cruise in 1998 (Fig. 1). Surface and thermocline waters of the two sites are linked by the MSTF and are connected with the western tropical Pacific and the eastern Indian Ocean via the ITF. The northern site, MD2178, is in the upstream of the ITF western route and can approximately represent the original characteristics of the ITF inflow, while the southern one, MD2161, is under the influence of both the MSTF and the SCSTF. The proxy-derived temperature and salinity of surface water and thermocline water from both sites were compared, which will enable us to assess the paleo-MSTF variability between the Holocene and the last glacial period.

## Results

### Age model

**Figure 2:**
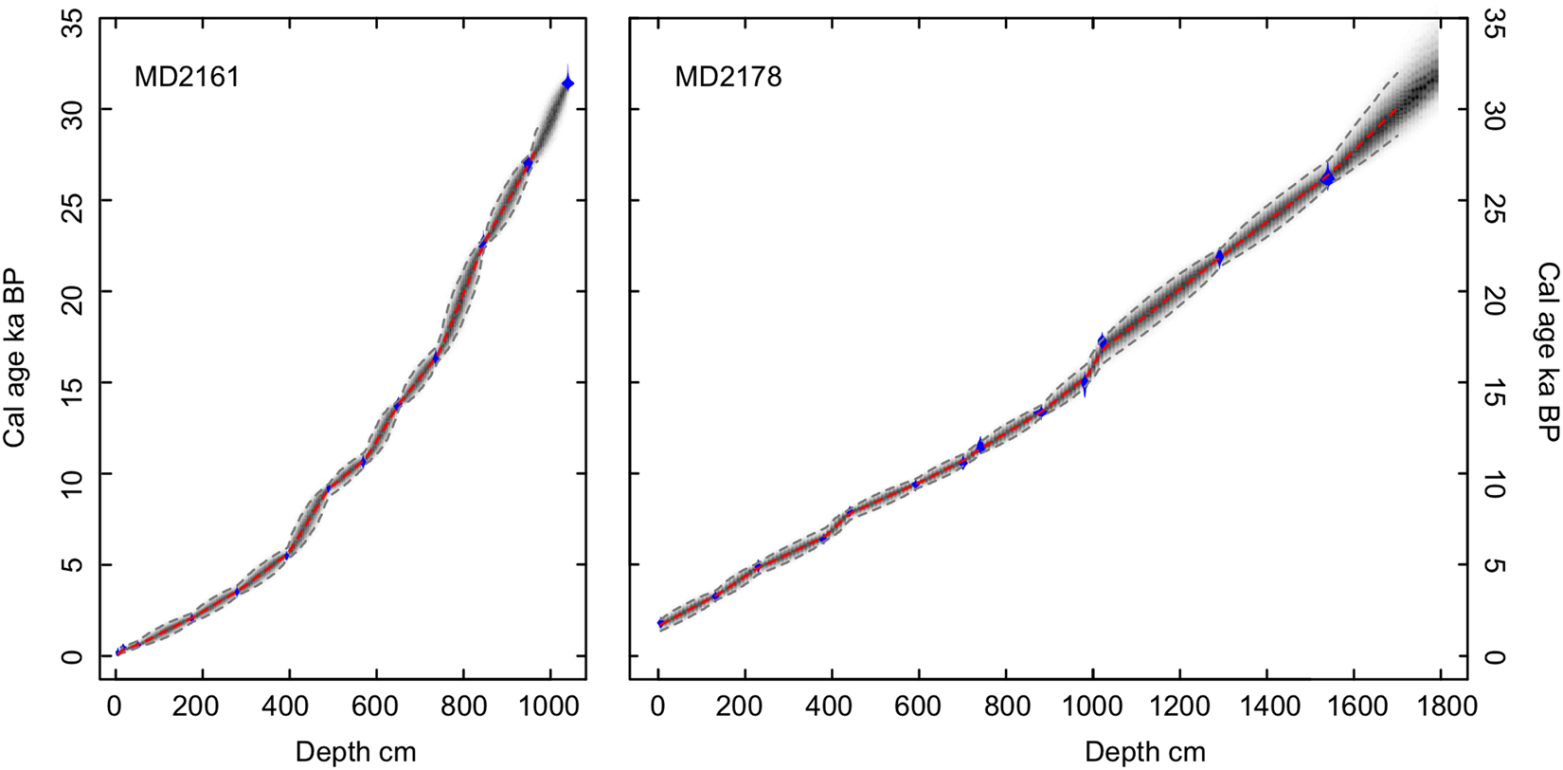
Age models of MD2161 (0–970 cm) and MD2178 (0–1610 cm) based radiocarbon data. Constant reservoir age correction of 400 years and the model is established using Bacon software^34^. The diagram shows age model control points (blue), the age-depth model with grey stippled lines of 95% confidence intervals, and red curve of a single “best” model based on the weighted mean age for each depth.

The age models of two cores are established on the basis of AMS^14^C measurements on planktonic foraminifera *Globigerinoides ruber* (sensu stricto) tests from 15 samples of MD2161 analyzed in *Laboratoire des Sciences du Climat et de l’Environment* (LSCE), France, and 14 samples of MD2178 in Leibniz Laboratory, Kiel University, respectively (Fig. 2 and Supplementary Table S1). Volcanic glass largely occurred at depth ~8 cm of the core MD2161 corresponds with the Tambora eruption in 1815 AD and is treated as an accurate age control point^33^. With local seawater reservoir age offset of ~7 ± 80 years relative to the global Marine13 curve, the age-depth model was established using Bacon, a Bayesian statistics software^34^ (Fig. 2). According to the age models, sediment of both cores deposited without any recognizable hiatus within the studied intervals, while the sediment of the past 1.6 ka for MD2178 is missing. The average sedimentation rates of both cores are greater than 30 cm/ka and the sample resolutions reach up to 40 yr and 60 yr for MD2161 and MD2178, respectively.

### Variability of the upper water temperature

**Figure 3:**
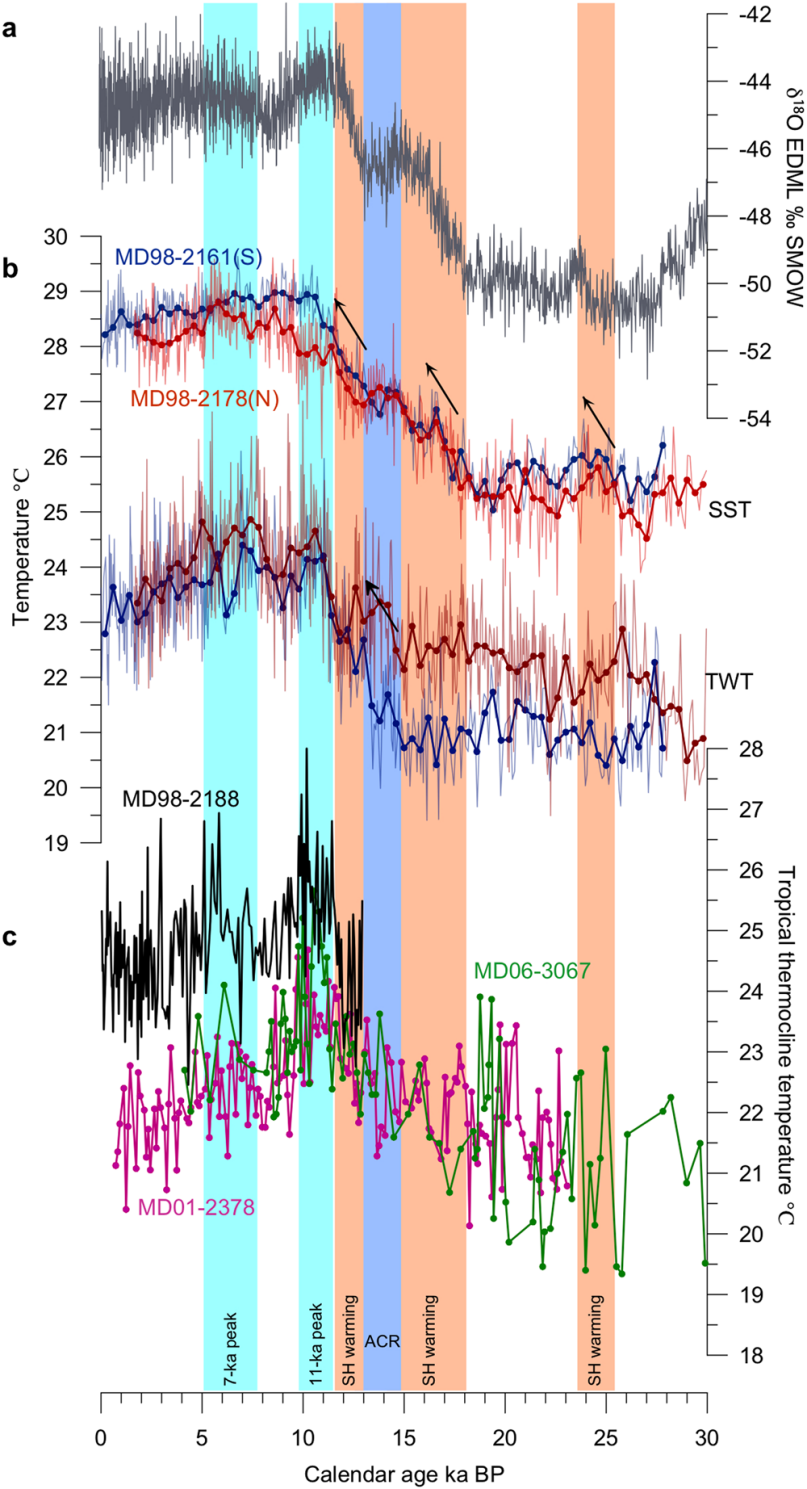
(**a**) Antarctic ice core δ^18^O from European Project for Ice Coring in Antarctica (EPICA) Dronning Maud Land^35^. (**b**) SST and TWT records of MD2161 (blue) and MD2178 (red) with 400-yr bin smoothing line (thick blue and red line), in which modern annual-mean SST and TWT values are denoted with black arrows. (**c**) Previous published TWT records from the IPWP region^30,37,42^, in which blue bars mark two TWT peaks at ~11 ka BP and ~7 ka BP. ACR and SH denote Antarctic Cold Reversal and Southern hemisphere, respectively.

The late Holocene (0~1 ka BP) sea surface temperature (SST; derived from the Mg/Ca of the mixed-layer-dwelling species of planktonic foraminifera *G. ruber*) of MD2161 is estimated to be 28.4 °C, 0.5 °C lower than the modern annual SST (Fig. 3b), but this discrepancy is within the error of temperature estimates. The LGM (18~23 ka BP) SST of this site is ~25.6 °C, 2.8 °C lower than the late Holocene level. Modern sediment of MD2178 is absent but the average SST over the Holocene is 0.5 °C lower than that of MD2161 (Fig. 3b). However, the modern annual SST of the northern site is slightly warmer or equal to that of the southern site. This inconsistence between the reconstruction and the modern situation is perhaps attributed to the differences in the depths and the average temperatures of their mixed layers (0~50 m) where *G. ruber* mainly dwells. On 50 m level of water depth today, the southern site is 0.65 °C warmer than the northern site due to its deeper mixing layer. During the last deglaciation, the SST records of two studied sites clearly show stepwise warming similar to the Antarctic ice core δ^18^O record^35^ (Fig. 3a).

The thermocline water temperature (TWT; derived from the Mg/Ca of the upper-thermocline-dwelling species of planktonic foraminifera *Pulleniatina obliquiloculata*) of MD2161 is around 21 °C during the last glacial period, cooler than the late Holocene level by ~2.5 °C (Fig. 3b). The TWT of MD2178 is slightly warmer than that of MD2161 during the Holocene (Fig. 3b), in consistent with the modern situation. But the discrepancies between the TWTs of both sites increase up to 1 °C on average during the last glacial period and reach the maxima during 13.4~19 ka BP and 24.2~27 ka BP. Both TWT records display stepwise warming during the 15~13 ka BP and the beginning of the Holocene. They reach the first striking peak at 10~11 ka BP(denoted as 11-ka peak hereafter) and another plateau-like one at 5~7.8 ka BP (denoted as 7-ka peak hereafter) with an obvious cooling between the two peaks and then continuously cooled after 5 ka BP.

**Figure 4:**
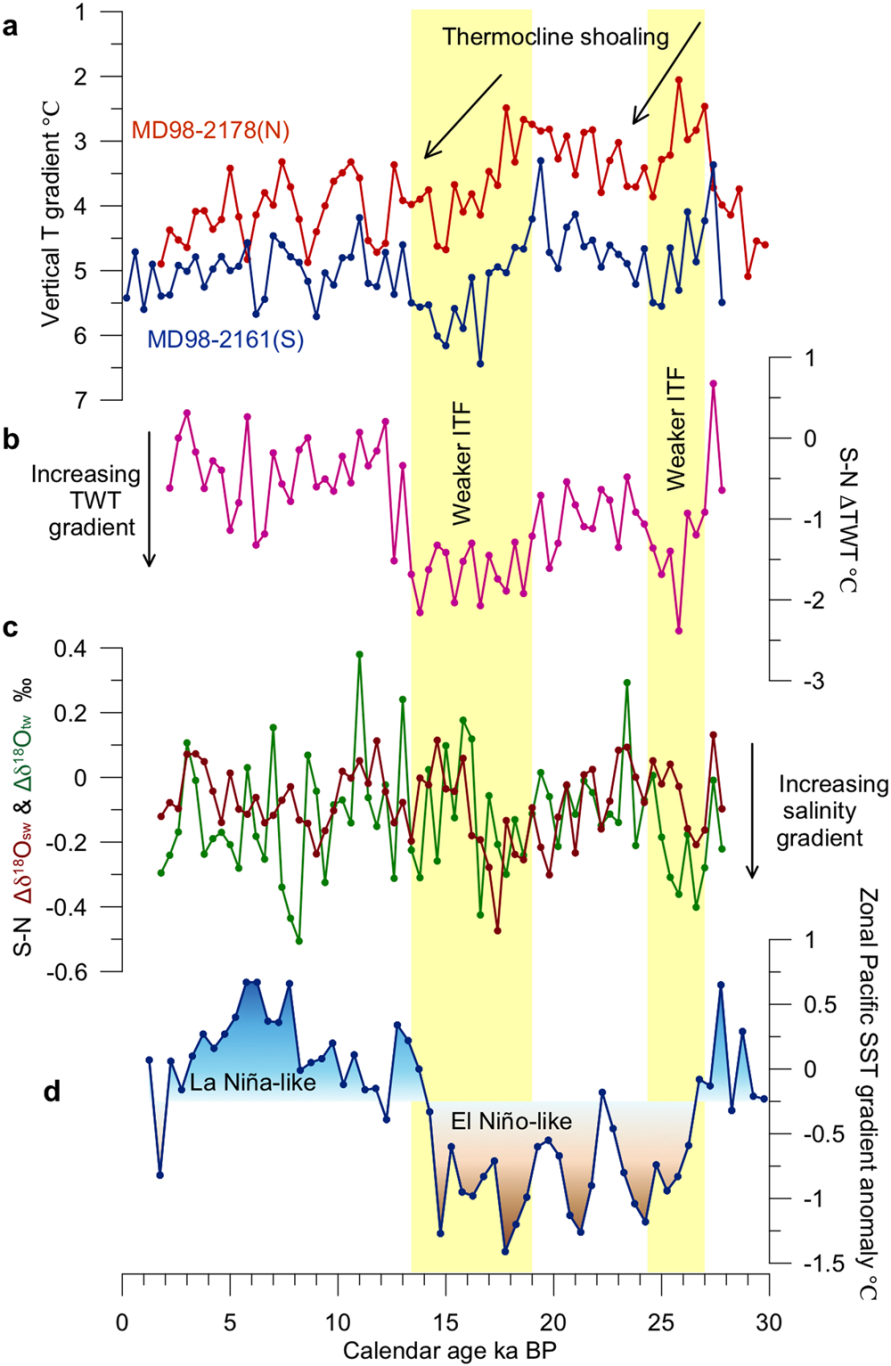
(**a**) Upper ocean temperature gradients (SST minus TWT) from MD2161 (blue) and MD2178 (red). (**b**) South–North thermocline temperature gradient (or ΔTWT_S−N_) across the Makassar Strait indicated by TWT differences between MD2161 and MD2178, with lower (higher) values indicating weaker (stronger) ITF and heat transport ability. Dashed horizontal lines mark average values of corresponding time periods. (**c**) Similar to (**b**) but for South–North δ^18^O gradients at the surface (Δδ^18^O_sw_; red) and thermocline (Δδ^18^O_tw_; green). (**d**) Paleo-ENSO-like mean state changes indicated by zonal SST gradient across the tropical Pacific (ref.^33^). Yellow-shaded bars mark two intervals of weakened ITF during 13.4~19 ka BP and 24.2~27 ka BP.

The difference between parallel-measured SST and TWT is taken as a proxy for upper-ocean thermal gradient. As is present in Fig. 4a, the upper-ocean thermal gradient at MD2178 is smaller than that at MD2161 both in the Holocene and the last glacial period. The former is smaller during the last glacial period relative to during the Holocene, while the latter is roughly comparable between the last glacial period and the Holocene.

### Seawater oxygen isotope

The *G. ruber* and *P. obliquiloculata* δ^18^O records of both sites together show typical glacial/interglacial changes (Supplementary Fig. S3). The S-N differences in the surface and thermocline water δ^18^O (denoted as ∆δ^18^O_sw_ and ∆δ^18^O_tw_, see ‘Methods’) between the two sites, which are used to indicate the surface salinity differences and those of the subsurface, respectively, are essentially similar both in millennial- and longer time scales (Fig. 4c). Clearly, both records indicate larger S-N salinity gradient during two intervals (15~18 ka BP and 25~27 ka BP) (Fig. 4c), implying a relative fresher southern Makassar Strait both in surface and thermocline.

## Discussion and Conclusions

It can been seen in Fig. 4 that the thermocline temperature and salinity gradient across the Makassar Strait increased during the last glacial period relative to during the Holocene and was significantly larger during 13.4~19 ka BP and 24.2~27 ka BP. Assuming the tidal mixing intensity of the Indonesian sea in a glacial boundary condition similar to the modern level, a likely explanation for the increased contrast of the thermocline hydrology across the Makassar Strait is related to the strength of MSTF, which determines how much warmer and saltier thermocline water is transported across the Strait and thus the S-N thermocline temperature or salinity gradients between MD2161 and MD2178. Responding to a short-term (e.g. seasonal) increase of the inflow of Pacific water, the thermocline hydrological gradient may be enhanced. But under an equilibrium state at multi-millennial scales, enhanced TWT and δ^18^O_tw_ gradients during 13.4~19 ka BP and 24.2~27 ka BP likely indicate weakened MSTF transport relative to the situation during the Holocene.

However, the vertical mixing intensity may vary between the Holocene and the last glacial period. The SE-monsoon induced oceanic upwelling and vertical mixing could result in a much cooler thermocline in the southern Makassar Strait during the last glacial period^29^. According to the vertical temperature gradient records of the two sites, the gradient is larger during the last glacial period relative to during the Holocene for MD2178 but is roughly comparable between the last glacial period and the Holocene for MD2161 (Fig. 4a). This implies that the vertical mixing or upwelling could have exerted some effect on the thermocline depth in the southern Makassar Strait. But the remarkably increased vertical temperature gradients of 13.4~19 ka BP and 24.2~27 ka BP at the two sites are hard to be explained by the monsoonal upwelling because there is a similar shoaling of the thermocline both at the north and the south off equator (Fig. 4a). Therefore, even if there is any impact of the SE-monsoon-induced upwelling or vertical mixing on the thermocline structure in the southern Makassar Strait, it could not completely explain the remarkable thermocline cooling there during the last glacial period (Fig. 3b).

Another possible mechanism for the glacial increased S-N TWT gradient (reflected by the decreased S-N ∆TWT values) may come from ENSO-like dynamical modulation of the tropical Pacific mean climate state. On the centennial to millennial timescales in the Holocene, a possible impact of the mean climate state fluctuation across the tropical Pacific on the MSTF variability has been proposed^33^. Here we suggest that this impact may also exist on longer time scales like the glacial-interglacial cycles. The ∆TWT and ∆δ^18^O_tw_ records of the MSTF are comparable to paleo-proxies of zonal SST gradient across the equatorial Pacific^36^. During the last glacial period, particularly in two intervals of 13.4~19 ka BP and 24.2~27 ka BP, the enhanced S-N thermocline hydrological gradient indicated a weaker MSTF, while the smaller zonal SST gradient reflected an El Niño-like mean state over the equatorial Pacific (Fig. 4d). During 13~2 ka BP, the zonal SST gradient is at least 1 °C larger than that during the last glacial period, indicating a more La Niña-like mean state that prefers relatively stronger MSTF as indicated by the decreased S-N TWT gradient.

This control of the Pacific ENSO-like mean state on the ITF between the last glacial period and the Holocene is also evidenced in upstream regions of the ITF such as the Mindanao Dome, for example, the thermocline was shoaled and the Mindanao Dome was intensified during the early period of the last deglacial period^37^ when a weaker ITF was revealed by our records. This is also consistent with the finding that a weakened ITF always appear with strengthened Mindanao Current under modern El Niño state^38^. A possible mechanism is that an enhanced Mindanao current may increase the transport of warm and saline north Pacific tropical water eastward into the Pacific North Equatorial Counter-current, thus less would be leaked westward into the ITF. This enhanced eastward transport of warm water under an El Niño-like state may further cause eastward propagation of the equatorial Pacific atmospheric convection centre during the last glacial period. Paleoceanographic reconstruction shows that the *G. ruber* δ^18^O gradient between the equator and 8°N in the central Pacific was enhanced during the last glacial period relative to the Holocene, indicating an enhanced salinity gradient due to increased precipitation on the equator^39^. Numerical simulations also provide lines of evidences for the mean state condition that may slashed the ITF during the glacial times, for example, the tropical atmospheric circulation under the LGM sea level conditions shows eastward propagation of the deep convection centre along the equator^40^, and during the HS1 period there is a southward ITCZ migration and weakened trade wind in the southeastern equatorial Pacific^41^.

In a mean state with stronger MSTF since 13 ka BP, both TWT records of MD2161 and MD2178 show peaks at ~11 ka BP and ~7 ka BP (Fig. 3b), which are also significant in the TWT records from cores MD2188 and MD3067 of the western tropical Pacific (Fig. 3c and site locations shown in Supplementary Fig. S1). These warming peaks are largely synchronous among these four sites within age uncertainties (Fig. 3). The TWT peaks in MD 2188 and MD3067 have been proposed to be derived from a teleconnection with the subtropical North Pacific via subsurface lateral advection and/or formation rate of the North Pacific Tropical Water^42^ and/or ultimately a basin-scale subsurface temperature signal originated from the southern Hemisphere^43,44^. The synchronous occurrence of these peaks within the Indonesian seas implies that the TWT signal of the tropical Pacific has been brought to the Sulawesi Sea and southern Makassar Strait by the ITF.

However, the 7-ka peaks of the TWT records of MD2178 and MD2161 are warmer than their 11-ka peaks, different from the situation that the 11-ka peak is the highest one in the tropical Pacific (e.g., MD3067) where the subsurface water of the ITF originates from. This may reflect a thermocline warming in the Sulawesi Sea after the reoccurrence of the SCSTF. The discussed two TWT peaks happened in a mean state of intensifying MSTF since 13 ka BP, but the 11-ka TWT peak occurred before the re-opening of the Karimata Strait at ~9.5 ka BP, while the 7-ka peak appeared after that. Modern observations have suggested that the less salty surface-water input towards the southern Makassar via the Karimata Strait may cause the ITF more subsurface-intensified and make the thermocline of the East Indian Ocean relatively cooler^13^ (reflected by the TWT record of MD01-2378 from Timor Sea)^31^. Therefore, it is speculated that the relative warmer subsurface water in the Sulawesi Sea and southern Makassar Strait relative to the open western tropical Pacific after 9.5 ka BP could be attributed to a decrease in heat output from the Sulawesi Sea caused by hindered surface warm flow after the re-occurrence of the SCSTF. This inference is highly supported by ocean general circulation model experiments with and without the SCSTF, which reproduce a warmer anomaly over the Sulawesi Sea-Makassar Strait area but cooler conditions in the southern Indonesian seas, including the Flores Sea and Banda Sea, relative to the scenario without the SCSTF^45^.

To summarize, we found that the variability of the ITF since the last glacial period was mainly reflected in the sub-surface water. The south-north gradients of thermocline temperature and salinity across the Makassar Strait increased during 13.4~19 ka BP and 24.2~27 ka BP relative to the Holocene conditions, indicating a weakened ITF at that time. The climate mean state changes in the tropical Pacific is proposed to be a predominant modulator on the ITF variability between the Holocene and the glacial period, when the weakened trade wind stress under a mean state of reduced zonal temperature gradient may result in the weaker MSTF. The subsurface MSTF might be enhanced after ~9.5 ka BP due to the reoccurrence of the SCSTF with the re-opened Karimata Strait, which may have caused a reduction in the heat flux from the Sulawesi Sea to the downstream regions and thus a warmer Sulawesi Sea but a cooler Timor Sea, relative to the scenario without the SCSTF.

## Methods

The present two cores were both sampled at 1 cm interval to recover high resolution records. For each sample, shells of *G. ruber* and *P. obliquiloculata* were analysed for both Mg/Ca and δ^18^O at the State Key Laboratory of Marine Geology, Tongji University, respectively. Mg/Ca analyses were made on an IRIS Advantage inductively coupled plasma–optical emission spectrometer with reproducibility of ±0.145 mmol/mol for *G. ruber* and ±0.148 mmol/mol for *P. obliquiloculata*, about 9.6% and 17.3% relative to the averages of the two cores, respectively. The pre-treatment procedures with a reductive cleaning step followed Martin & Lea (2002)^46^ and additional checking and removing of potential contamination were performed under a microscope particularly for *P. obliquiloculata*. δ^18^O of the two species was measured on a Finnigan-MAT253 mass spectrometer, following the method described by Cheng *et al*.^47^. Conversion to the international Peedee Belemnite (PDB) scale was performed using NBS19 standard, and the standard deviation is better than 0.05%.

The Mg/Ca of the surface dweller *G. ruber*, is transferred into SSTs using the calibration equation developed by Anand *et al*.^48^: Mg/Ca = 0.38 (±0.02) exp 0.090 (±0.003) T, which has been widely used for SST reconstruction of the tropical Pacific and Indian Oceans^30,37,42,49^. The TWT is estimated from Mg/Ca of the thermocline dweller *P. obliquiloculata* by using the species-specific calibration equation^48^: Mg/Ca = 0.328 (±0.007) exp 0.090 (±0.003) T. These two calibrations are both established for Mg/Ca measurements on samples cleaned by the non-reductive method. Therefore, the dissolution effect by the reductive cleaning step on both species was corrected before temperature estimations. According to contrast experiments between cleaning methods with- and without-reduction, Mg/Ca in *P. obliquiloculata* shells decrease in average by ~6.0% when the reduction step was added. This could be regarded as a conservative estimate because *G. ruber* should be more sensitive to reductive dissolution than *P. obliquiloculata*.

Because the δ^18^O of foraminiferal calcite shell includes both the seawater δ^18^O information and the temperature effect, the latter reconstructed by Mg/Ca proxy is subtracted to calculate the surface and thermocline seawater δ^18^O values (marked with δ^18^O_sw_ and δ^18^O_tw_, respectively), using the equation^50^: T (°C) = 16.9 + 4.38 (δ^18^O_c_ − δ^18^O_sw_) + 0.1(δ^18^O_c_ − δ^18^O_sw_)^2^, with a correction of −0.20‰ applied to the seawater oxygen isotope δ^18^O_sw_ from standard mean ocean water (SMOW) to PDB standard. Due to the rather large error and uncertainties hidden in the estimated sea-water δ^18^O, here only the differences between the two sites are used to indicate relative salinity gradient across the Makassar Strait both in the surface and thermocline. In this way, the global ice volume and regional hydrological climate effects may be largely subtracted and these gradients are likely caused by the local oceanography dynamics (e.g. upwelling, mixing and current). To reduce all kind of errors potentially induced by the proxy estimates and age models, we calculated the binning average values with a window of 400 years for the δ^18^O_sw_ and δ^18^O_tw_ records.

### Data availability

All data generated or analysed during this study are included in this published article (and its Supplementary Information files). The complete dataset of this study is available on mlab.tongji.edu.cn or upon request from jian@tongji.edu.cn.

## Electronic supplementary material

Supplementary Information
