# Supplementary material for: Variability of the Indonesian Throughflow in the Makassar Strait over the Last 30 ka
Source: Sci Rep. 2018 Apr 9;8:5678. doi: 10.1038/s41598-018-24055-1 (PMC5890278; doi:10.1038/s41598-018-24055-1)
Supplement: Supplementary file 1 — Supplementary Information [file 41598_2018_24055_MOESM1_ESM.pdf]

Supplementary Information for

Variability of the Indonesian Throughflow in the Makassar Strait over the Last 30 ka

Weijia Fan[1,2], Zhimin Jian[2 *], Zhihui Chu[2,4], Haowen Dang[2], Yue Wang[2], Franck Bassinot[3], Xiqiu Han[1], Yeping Bian[1,2]

[1] Key Laboratory of Submarine Geosciences, Second Institute of Oceanography, State Oceanic Administration, Hangzhou 310012, China

[2] State Key Laboratory of Marine Geology, Tongji University, Shanghai 200092, China

[3] Laboratoire des Sciences du Climat et de l'Environnement, UMR 8212 CEA-CNRS-UVSQ (LSCE), Gif-sur-Yvette 91198, France

[4] Shanghai Tech University, Shanghai 201210, China

* Corresponding author. Fax: +86 21 65988808; E-mail address: jian@tongji.edu.cn

Contents of this file

Table S1
Figures S1 to S3

References

1 Fan, W., Jian, Z., Bassinot, F. & Chu, Z. Holocene centennial-scale changes of the Indonesian and South China Sea throughflows: Evidences from the Makassar Strait. Global and Planetary Change 111, 111-117, doi:10.1016/j.gloplacha.2013.08.017 (2013).

2 Amante, C., Eakins, B.W. ETOPO1 1 Arc-Minute Global Relief Model: Procedures, Data Sources and Analysis. NOAA Technical Memorandum NESDIS NGDC-24. National Geophysical Data Center, NOAA, doi:10.7289/V5C8276M. <https://ngdc.noaa.gov/mgg/global/global.html > (2009)

3 Locarnini, R. A. et al. World Ocean Atlas 2013, Volume 1: Temperature in NOAA Atlas NESDIS 73 (e.d. Levitus, S., Technical e.d. Mishonov, A.) 40 pp (Silver Spring, 2013).
4 Schlitzer, R. Ocean Data View. <http://odv.awi.de.> (2017).

Table S1 Calibration of the AMS^{14}C measurements

MD98-2161[a]		MD98-2178[b]	
Depth [cm]	^{14}C age [ya BP]	Depth [cm]	^{14}C age [ya BP]
0~10[c]	560±60	6[c]	2210±21
17[c]	855±55	132[c]	3400±40
51[c]	1030±03	230[c]	4665±66
175[c]	2415±41	381[c]	5965±96
279[c]	3615±61	441[c]	7470±47
393[c]	5135±13	592[c]	8745±74
489[c]	8595±59	701[c]	9675±67
569[c]	9720±72	741[c]	10370±03
648[c]	12255±22	881[c]	11885±18
650[c]	12380±23	981	13040±30
736	13955±39	1021	14580±45
844	19020±90	1291	18470±847
846	19310±93	1540	22340±234
948	23060±30	1790	34470±447
1040	28090±809		

[a] AMS^{14}C analyses provided by LSCE in France.

[b] Provided by Leibniz Laboratory, Kiel University, Germany.

[c] Data published in reference No. 1 of this supplementary file.

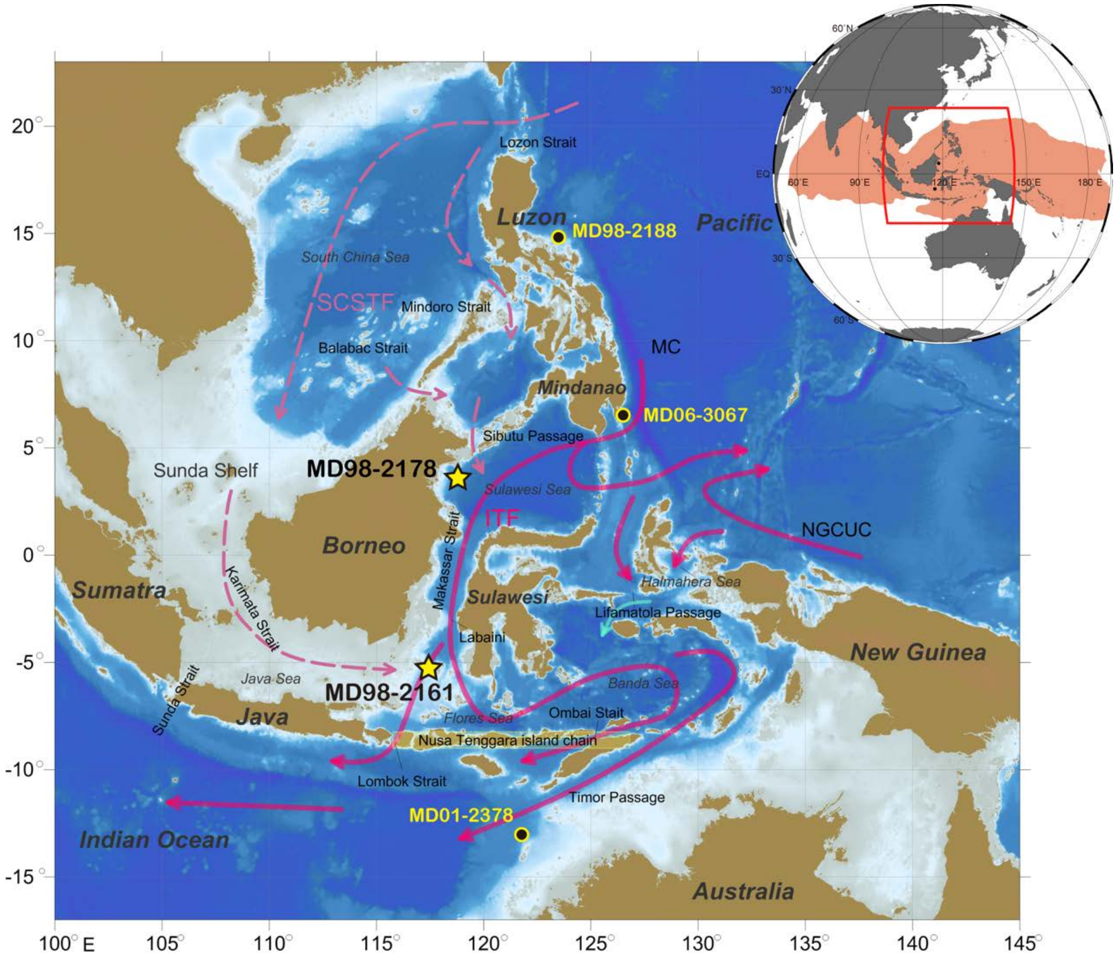

Figure S1. Location of the study cores (yellow pentagrams) and those previously studied sites mentioned in the paper (black dots). Shaded red in the index map indicates the West Pacific Warm Pool and the red unfilled polygon highlights the main region studied in this paper. The routes of the ITF (red arrows) and the SCSTF (pink and dashed arrows) are sketched in the map. The base map is generated by purchased ArcGIS Desktop 10.2 software with the relief data of ETOPO1[2].

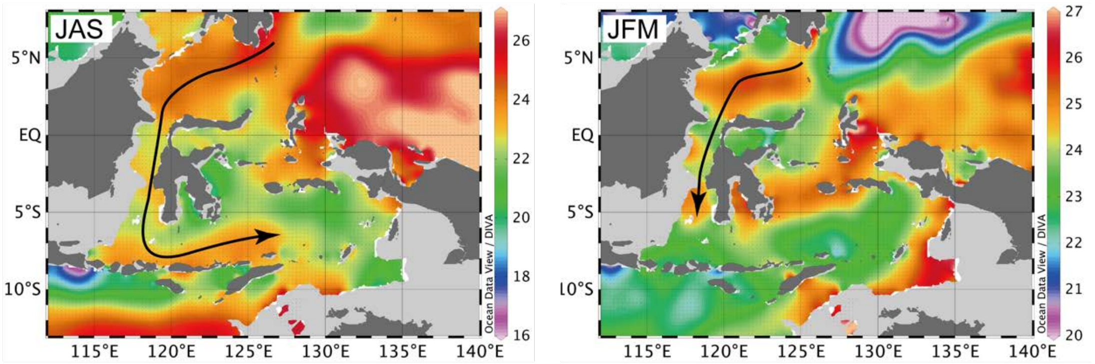

Figure S2. Seasonal temperature characteristics on 100 m level of water depth. It is observed that more subsurface warm water from the western Pacific are conveyed into the Indonesian Sea during July–September (JAS) due to stronger ITF than during January–March (JFM). The hydrological data are all derived from World Ocean Atlas 2013[3] and are displayed using Ocean Data View version 4.7.3[4].

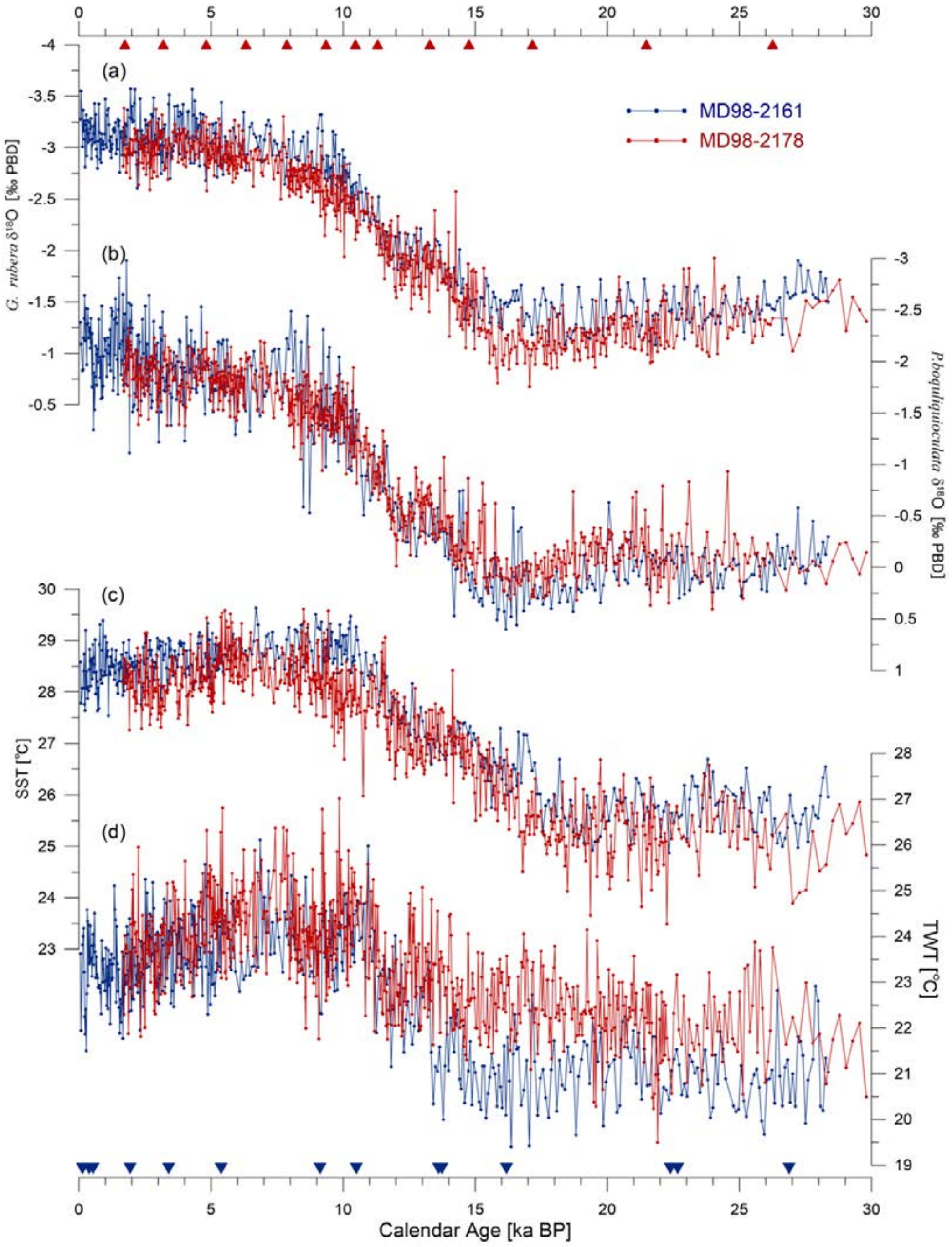

Figure S3. (a) *G. ruber* $\delta^{18}O$ records of MD2161 and MD2178. (b) *P. obliquiloculata* $\delta^{18}O$ records of the two cores. (c) SST records estimates from *G. ruber* Mg/Ca and (d) TWT records calculated from *P. obliquiloculata* Mg/Ca. The AMS^{14}C determinations are marked with triangles. Here, all the records from MD2161 are in blue color while those of MD2178 are in red color.
